# Rejection Sensitivity, Self-Compassion, and Aggressive Behavior: The Role of Borderline Features as a Mediator

**DOI:** 10.3389/fpsyg.2020.00044

**Published:** 2020-01-24

**Authors:** Eliane Sommerfeld, Mally Shechory Bitton

**Affiliations:** ^1^Department of Psychology, Ariel University, Ariel, Israel; ^2^Department of Criminology, Ariel University, Ariel, Israel

**Keywords:** borderline, rejection-sensitivity, self-compassion, aggressive behavior, personality and behavior

## Abstract

This study used mediation analyses to examine the assumption that the presence of borderline personality features mediates the relationship between rejection sensitivity (RS), self-compassion, and aggressive behavior. Sixty adults consisting of 31 participants diagnosed with borderline personality disorder and 29 participants with no diagnosis of borderline personality disorder were assessed for RS, self-compassion, aggressive behavior, and borderline personality features. Mediation was found for the total aggression score, anger score, and hostility score regarding both self-compassion and RS. Mediation was also found regarding RS and verbal and physical aggression, and regarding self-compassion and verbal and physical aggression. These findings provide evidence that the presence of borderline personality features is an important factor in explaining the associations linking RS and self-compassion to aggressive behavior. High RS and low self-compassion are associated with greater borderline personality features, which in turn relate to increased aggressive behavior.

## Introduction

Interpersonal aggression is closely dependent on the ways people perceive and interpret themselves and others in interpersonal situations. Rejection sensitivity (RS) and self-compassion are two constructs that capture the way that individuals feel in relation to themselves and to others and are presumed to be related to aggressive behavior (e.g., Ayduk et al., 1999; Downey et al., 2000; Morley, 2015). Borderline personality disorder is characterized by proneness to interpersonal hostility and aggression (Sansone and Sansone, 2012; American Psychiatric Association [APA], 2013). However, investigation into the relationship between RS, self-compassion, and aggressive behavior in the context of borderline personality features (i.e., borderline cognition and emotions) has yet to be adequately studied. The aim of the present study was to investigate the associations between RS and self-compassion and aggressive behavior, as well as to explore the role of borderline personality features as a mediator of these associations.

### Rejection Sensitivity and Aggressive Behavior

Interpersonal rejection is associated with aggressive behavior (Leary et al., 2006; Lawson and Brossart, 2013). However, people differ in the extent to which they subjectively *feel* rejected in various situations and thus in the ways they respond to these experiences. RS is defined as a cognitive–affective processing disposition (Downey and Feldman, 1996) that leads one to “anxiously expect, readily perceive and intensely react to rejection” (Downey et al., 2004, p. 668). It is assumed that this heightened sensitivity to rejection is a developmental outcome of prolonged and severe rejection of a child by significant others (Bowlby, 1980; Feldman and Downey, 1994).

People with high RS are hypervigilant for rejection and tend to perceive ambiguous negative behavior as a sign of rejecting (Downey and Feldman, 1996). They also tend to blame others, feel hurt or angry, and respond by withdrawal or aggression. For example, Downey et al. (2000) examined the association between rejection expectations and intimate violence. They found that young men who reported relatively high investment in romantic relationships and had anxious expectations of rejection reported higher rates of dating violence. In three other studies (Ayduk et al., 1999), women who had elevated levels of RS displayed stronger hostile reactions to rejection. That is, individuals with elevated RS tend to not only readily perceive rejection, but also overreact to it.

### Self-Compassion and Aggressive Behavior

Self-compassion is a healthy self-attitude characterized by being kind and non-judgmental toward oneself, recognizing one’s experiences as common to humankind, and being mindfully aware of one’s failings and shortcomings (Neff, 2003a). When individuals with high levels of self-compassion experience failure, they have a kind, accepting, and understanding stance toward themselves. Self-compassion was found to be associated with different measures of mental health (Barnard and Curry, 2011; Zessin et al., 2015).

Although self-compassion is more related to the way people treat themselves, studies have also shown its effect on interpersonal well-being. Indeed, several studies have found that self-compassion is associated with interpersonal closeness and social connectedness, concern for others, and relational well-being (Neff, 2003a; Neff et al., 2007; Neff and Pommier, 2013; Yarnell and Neff, 2013). Self-compassion is also associated with violent criminality (for a recent review, see Morley, 2015). However, only a few studies have directly addressed the possible negative association between self-compassion and aggressive behavior. For example, in one study (Neff and Vonk, 2009) self-compassion was found to be negatively related to anger in various social situations. In a sample of adolescent drop-outs, self-compassion was negatively related to aggression (Barry et al., 2015). In addition, in the context of couples (Neff and Beretvas, 2013), individuals with higher self-compassion were found to be perceived by their partners as being less controlling and aggressive.

### Borderline Personality Features as Mediator of Aggressive Behavior

Borderline personality disorder is associated with impairments in both self and other perception and emotional dysregulation. According to the DSM-5 (American Psychiatric Association [APA], 2013), one feature of individuals with borderline personality disorder is their tendency to experience inappropriate and intense anger. They may frequently display verbal or physical aggression due to difficulties in modulating or controlling their anger responses. According to the alternative model of personality disorder in the DSM-5 (American Psychiatric Association [APA], 2013), borderline personality disorder is often associated with significant impairments in interpersonal functioning manifested by limitations in recognizing the needs and feelings of others, as well as difficulties with trust and intimacy issues. Their pathological personality traits consist of negative affectivity, antagonism, and disinhibition.

Borderline personality features, i.e., cognitions and emotions associated with borderline personality disorder, were tested as a mediator variable in the context of aggressive behaviors toward self and others. For example, borderline personality features were found to be a mediator in the relationship between child maltreatment and adult suicide potential among students (Allen et al., 2013), and between attachment and intimate partner violence (Mauricio et al., 2007; Lawson and Brossart, 2013).

### The Present Study

**FIGURE 1:**
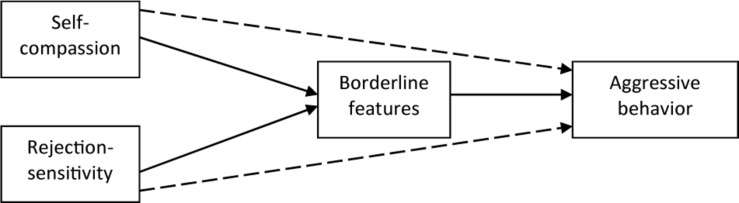
Proposed model of aggressive behavior. If a mediation effect occurs, the broken lines which represent direct links between the predicting and the predicted variables are annulled or reduced.

Borderline personality is already known to be associated with lower self-compassion, higher RS, and higher aggression. However, the causal direction of these relationships remains unclear. One alternative is that borderline personality features are formed earlier in a child’s life, contribute later on to the development of RS, and lower self-compassion, which then lead to heighted aggression in interpersonal relations. According to this supposition, borderline personality predicts aggression due to heightened RS and lower self-compassion. Another alternative is that RS and lower self-compassion are basic personality features that advance the development of a fully configured BPD and consequently of a tendency toward aggression in interpersonal relations. This later proposition is consistent with psychoanalytic theoretical ideas suggesting that a healthy personality development depends on sensitive attention to the needs of the child, and on the experience of primary caregivers as providing mirroring, acceptance, and admiration (e.g., Kohut, 1977). Accordingly, negative experiences in this context may subsequently lead to various personality dysfunctions and borderline characteristics (for a review, see Bradley and Westen, 2005). This assumption is compatible with a more dimensional view of borderline personality disorder. On this basis and in according to prior findings regarding the mediating role of borderline features (i.e., Mauricio et al., 2007; Allen et al., 2013; Lawson and Brossart, 2013), our assumption in the present study was that borderline features would mediate the relationship between RS and aggressive behavior and the relationship between self-compassion and aggressive behavior (Figure 1). However, since both alternatives make sense, we adopted a competing model strategy (Hair et al., 1995) in which a proposed mode is tested in comparison to an alternative model, with the same data. Following this strategy, we tested a competing model in which RS and self-compassion were located as mediators in the association between borderline personality features and aggression.

Mediation involves a predictor that affects a dependent variable indirectly through a mediating variable, namely a mediator (Baron and Kenny, 1986). Mediation effects are assumed to be present when three conditions are met: (a) a significant relationship exists between the predictor and the dependent variable; (b) a significant relationship exists between the predictor and the mediator; and (c) the relationship between the predictor and the dependent variable is annulled or reduced in the presence of the mediator. In the present study, these conditions were tested. Specifically, an attempt was made to explore whether the relationship between self-compassion and rejection-sensitivity (the predictors) and aggressive behavior (the dependent variable) is mediated by borderline personality features (the mediator).

Our hypotheses were as follows: (a) self-compassion, rejection-sensitivity, and borderline personality features are associated with aggressive behavior; (b) self-compassion and RS are associated with aggressive behavior; and (c) the direct relationship between self-compassion and aggressive behavior and the direct relationship between RS and aggressive behavior are reduced in the presence of borderline personality features. The model was tested separately for the total score of aggressive behavior, as well as for the different components of aggressive behavior: i.e., verbal aggression, physical aggression, anger, and hostility.

## Materials and Methods

### Participants and Procedure

The study included 60 adults (40% male and 60% female) (age mean = 26.41, SD = 6.18). The first group included 31 participants, who were members in a support group for people suffering from borderline personality disorder, after being formally diagnosed with this condition by a psychiatrist. The second group included 29 participants with no psychiatric history, who were sampled from the community. Most of the participants were single (80%), while the others were married (13.3%) or divorced (6.7%). Only seven participants (11.7%) had children. Almost all participants (95%) reported being secular. About half of them (51.7%) had a high school education, with the others (48.3%) reporting higher academic education.

The study was approved by the institutional review board. The data were gathered by means of a contact person who served as a mediator between the researchers and a support group of people diagnosed as suffering from a borderline disorder. The contact person approached people in the support group personally and asked whether they would be willing to participate in the study. Potential participants were screened by being asked to report all their known psychiatric diagnoses, as formally diagnosed by a psychiatrist, as well as the circumstances of receiving the psychiatric diagnoses. The inclusion criterion was reporting of having been diagnosed with borderline personality disorder. It should be noted that in our country the diagnosis of borderline personality disorder is conducted on the basis of the DSM criteria. Exclusion criterion was reporting of having been diagnosed with a psychotic spectrum disorder. After signing an informed consent form, the questionnaire was sent to them by e-mail. All participants received an explanation of the study and the questionnaire was sent by e-mail to the 36 people who gave their consent. Thirty-one fully completed questionnaires were returned. After gathering the data of the trial group, and in order to reach as similar a sample as possible with regard to demographic features (for instance, age and gender), a notice was posted at the university seeking suitable respondents. Potential participants were screened and included only if they reported not suffering from any personality disorder or other psychiatric problem and never experiencing recourse to psychiatric services., Both groups were matched by age and gender. The differences between the borderline group (32.3% male, 67.7% female; aged 19–44 years, *M* = 26.8, SD = 5.9) and the control group (48.3% male, 51.7% female; aged 21–54 years, *M* = 25.9, SD = 6.6), were found to be non-significant for age, *t*(58) = 0.60, *p* > 0.05, and for gender, χ^2^ = 1.60, df = 1, *p* > 0.05.

### Instruments

*The Questionnaire of Thoughts and Feelings* (QTF) (Renneberg et al., 2005) is a self-administered questionnaire that assesses feelings, cognitions, and assumptions characteristic of borderline personality disorder (e.g., “I hate myself,” “Intimate relationships are threatening,” and “Sometimes I want to hurt myself”). It is not intended to be a diagnostic instrument of borderline personality disorder, but to assess cognitions that are characteristic of borderline personality disorder, based on cognitive and bio-social theories of borderline personality disorder. The instrument was used in clinical (Ayduk et al., 2008) as well as non-clinical samples (Rosenbach and Renneberg, 2014). Each item is rated on a 5-point scale, and the mean score indicates the self-reported level of borderline specific cognitions and feelings. Previous studies have demonstrated its reliability and validity for borderline personality disorder (e.g., Renneberg et al., 2005; In-Albon et al., 2008). In the current study, the reliability index was α = 0.94.

The *Aggression Questionnaire* (AQ; Buss and Perry, 1992) is a revision of the Buss–Durkee Hostility Inventory (Buss and Durkee, 1957). It is a 29-item instrument that records self-reported feelings and behaviors. It consists of four subscales: physical aggression, verbal aggression, anger, and hostility. It is an extensively used psychometrically validated measure and has been shown to reliably differentiate aggressive individuals in normal, forensic, and clinical samples (e.g., Shechory et al., 2013; Carver, 2014). In the present study, the reliability indices were α = 0.71 for the verbal aggression subscale, α = 0.67 for the anger subscale, α = 0.80 for the physical aggression subscale, α = 0.85 for the hostility subscale, and α = 0.92 for the total aggression score.

The *RS Questionnaire* (Downey et al., 1998) assesses anxious expectations for rejection by significant others (Downey and Feldman, 1996). Participants are presented with nine situations that might result in rejection and are asked to rate how much concern or anxiety they would feel at the possibility of rejection, as well as how likely the rejection would be. Ratings are conducted on a 6-point scale (1 = “very unconcerned/unlikely;” 6 = “very concerned/likely”). In order to calculate the total score for each situation, the anxiety score is multiplied by the likelihood score, and then the mean is computed. Previous studies have demonstrated the measure’s convergent, discriminant, and behavioral validity (e.g., Downey and Feldman, 1996; Berenson et al., 2009). In the present study, the reliability index was α = 0.86.

The *Self-Compassion Scale* (Neff, 2003b) is a 26-item scale, which assesses the three components of self-compassion: Self-kindness versus self-judgment; common humanity versus isolation; and mindfulness versus over-identification. Participants are asked to rate each item on a five-point scale ranging from 1 (almost never) to 5 (almost always). Although it is possible to calculate six mean scores for the various dimensions of the scale, for the present study we calculated only the total score due to the limited sample size. Evidence for the reliability and validity of the scale was presented in a series of studies (Neff, 2003b, 2016). Reliability indices in the current study were: α = 0.89 for the self-kindness subscale, α = 0.87 for the self-judgment subscale, α = 0.81 for the common humanity subscale, α = 0.84 for the isolation subscale, α = 0.80 for the mindfulness subscale, α = 0.78 for the over-identified subscale, and α = 0.95 for the total scale.

### Data Analysis

We used SPSS version 21 (IBM Corp, 2012) for conducting MANOVA in order to perform a preliminary comparison between participants in the self-defined borderline group and controls on all research variables. Mediation analysis was conducted with the Preacher and Hayes (2004) procedure with bootstrapping. As group differences in aggression were significant, mediation analyses were conducted on the whole sample while controlling for group (1 = borderline, 0 = control). Age, gender, place of birth, education, and economic status were unrelated with aggression scores. Differences in aggression were significant only by work status (1 = partial and full, 0 = not working): for working participants, *M* = 3.99 *SD* = 0.98 and for non-working participants, *M* = 3.04 *SD* = 0.92 [*t*(58) = 3.68, *p* < 0.001]. Therefore, analyses were controlled for work status. Variables were standardized.

## Results

### Preliminary Analyses: Comparing the Research Variables Between Groups

The multivariate analysis revealed that the differences between participants with borderline personality disorder and controls were overall significant, *F*(13,46) = 3.77, *p* < 0.001, partial eta squared = 0.52. The univariate analyses are presented in Table 1. All variables, except verbal aggression, differed significantly between groups. Participants with borderline personality disorder reported higher levels of aggressive behavior, borderline features, RS, and lower levels of self-compassion compared to controls.

**TABLE 1:** Differences between groups in the study variables.

Variable	Borderline personality disorder group (*N* = 31), mean (SD)	Control group (*N* = 29), mean (SD)	*F*	*p*	$\eta_{p}^{2}$
Borderline features	3.44 (0.91)	2.00 (0.63)	49.79	0.000	0.46
**Violence**					
Verbal	4.15 (1.35)	3.77 (0.96)	1.64	0.21	0.03
Anger	4.12 (1.14)	3.16 (0.89)	13.09	0.001	0.18
Physical	3.61 (1.18)	2.84 (1.00)	7.52	0.008	0.12
Hostility	4.53 (1.18)	3.31 (1.18)	16.04	0.000	0.22
Total	4.08 (1.07)	3.21 (0.85)	12.20	0.001	0.17
Rejection sensitivity	14.67 (6.16)	9.05 (2.98)	19.75	0.000	0.25
**Self-compassion**					
Self-judgment	3.94 (0.93)	2.97 (0.91)	16.83	0.000	0.23
Self-kindness	2.11 (1.04)	2.95 (0.96)	10.54	0.002	0.15
Common humanity	2.21 (0.87)	3.14 (0.92)	16.07	0.000	0.22
Isolation	4.07 (0.80)	3.07 (1.04)	17.68	0.000	0.23
Mindfulness	2.26 (0.92)	3.24 (0.81)	19.26	0.000	0.25
Over-identification	4.19 (0.72)	3.20 (0.85)	24.16	0.000	0.29
Total score	2.14 (0.73)	3.03 (0.66)	24.61	0.000	0.30

### Mediation Analyses: Testing Borderline Features as a Mediator Variable

Table 2 presents the correlations between pairs of variables in the proposed model. As can be seen, all correlations were found to be significant. Specifically, higher levels of rejection-sensitivity and lower levels of self-compassion were significantly correlated with aggressive behavior.

**TABLE 2:** Correlation matrix for variables used in the path analysis (*N* = 60).

	Aggression (total score)	Borderline features	Rejection sensitivity	Self-compassion (total score)
Aggression (total score)	1.00			
Borderline features	0.67***	1.00		
Rejection sensitivity	0.42**	0.72***	1.00	
Self-compassion (total score)	−0.45***	−0.75***	−0.62***	1.00

** *p* < 0.01; *** *p* < 0.001.

**FIGURE 2:**
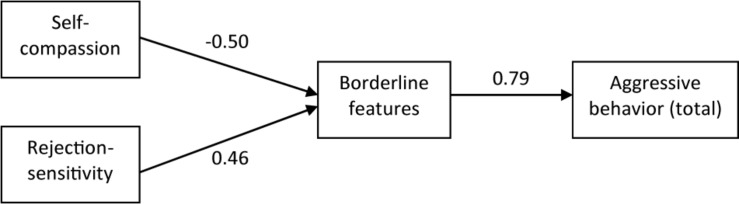
Final model of aggressive behavior predicted by self-compassion, rejection sensitivity, and borderline personality features.

The results of the mediation analyses are presented in Table 3 and Figure 2.

**TABLE 3:** Path coefficients and indirect effects for the mediation models (*N* = 60).

Dependent variable (DV)	Variable	Path coefficients		Indirect effects		
		To DV estimate (SE)	To mediator estimate (SE)	Estimate (SE)	Z	95% CI
Aggressive behavior (total)	Self-compassion	0.174 (0.156)	−0.503(0.096)***	−0.402(0.110)	3.664***	−0.658, −0.219
	Borderline features	0.799(0.178)***				
Aggressive behavior (total)	Rejection sensitivity	−0.148(0.146)	0.460(0.093)***	0.360 (0.117)	3.084**	0.159,0.612
	Borderline features	0.783(0.175)***				
Verbal aggression	Self-compassion	0.635(0.206)**	−0.503(0.096)***	−0.500(0.148)	3.383***	−0.821, −0.236
	Borderline features	0.994(0.235)***				
Verbal aggression	Rejection sensitivity	−0.120(0.208)	0.460(0.093)***	0.301 (0.125)	2.405*	0.097,0.579
	Borderline features	0.656(0.249)*				
Anger	Self-compassion	−0.098(0.176)	−0.503(0.096)***	−0.227(0.095)	2.384*	−0.408, −0.034
	Borderline features	0.451(0.201)*				
Anger	Rejection sensitivity	−0.114(0.164)	0.460(0.093)***	0.272 (0.101)	2.702**	0.102,0.493
	Borderline features	0.591(0.197)**				
Physical aggression	Self-compassion	0.392(0.176)*	−0.503(0.096)***	−0.504(0.122)	4.135***	−0.761, −0.287
	Borderline features	1.001(0.201)***				
Physical aggression	Rejection sensitivity	−0.177(0.170)	0.460(0.093)***	0.396 (0.122)	3.240**	0.189,0.669
	Borderline features	0.861(0.203)**				
Hostility	Self-compassion	−0.122(0.196)	−0.503(0.096)***	−0.380(0.143)	2.655**	−0.670, −0.141
	Borderline features	0.755(0.224)**				
Hostility	Rejection sensitivity	−0.161(0.182)	0.460(0.093)***	0.433 (0.167)	2.593**	0.154,0.809
	Borderline features	0.941(0.219)***				

* *p* < 0.05; ** *p* < 0.01; *** *p* < 0.001.

As can be seen, all indirect effects are significant. Self-compassion was negatively related with borderline cognitions, which were then positively related with aggression. RS was positively related with borderline cognitions, which were then positively related with aggression. Mediation was found for the total aggression score, anger score, and hostility score regarding both self-compassion and RS. Mediation was also found regarding RS and verbal and physical aggression, as well as regarding self-compassion and verbal and physical aggression.

In the context of a competing model strategy (Hair et al., 1995), in which a suggested model is compared with an alternative model using the same data, we compared our proposed model with a competing model. We did so with an alternative model in which borderline personality features were located as the predictor, the dependent variable was the total score of aggressive behavior, and self-compassion and RS were used as mediators. This model has proven non-significant (for self-compassion: indirect effect = −0.114, SE = 0.106, *Z* = 1.078, *p* = 0.281, 95% CI = −0.336, 0.086; for RS: indirect effect = 0.098, SE = 0.099, *Z* = 0.979, *p* = 0.328, 95% CI = −0.309, 0.092).

## Discussion

In the present study, we found that individuals who are highly sensitive to interpersonal rejection and who are less compassionate toward themselves tend to be more aggressive in their social relations. These findings are compatible with former studies that found a rejection–aggression link in children (e.g., Reijntjes et al., 2011) and adults (Leary et al., 2006; DeWall et al., 2009), as well as other studies that validated the relevance of RS as a mediator of this link (e.g., Downey et al., 1998; Ayduk et al., 2008). In relation to self-compassion, our finding fits other studies that have found a negative correlation between self-compassion and aggression (e.g., Neff and Beretvas, 2013; Barry et al., 2015).

In addition to the direct associations, our findings show that the relationships between RS and self-compassion and aggressive behaviors are mediated by borderline personality features. These findings add knowledge regarding the role of borderline personality features as a mediator in the relationship between RS, self-compassion, and aggressive behavior. They are consistent with other studies in which borderline personality was found to mediate the association between attachment insecurities and partner violence (Mauricio et al., 2007; Lawson and Brossart, 2013).

Regarding RS, borderline personality features mediated its association with the total aggression score, anger score, hostility, and verbal and physical aggression. Regarding self-compassion, borderline personality features mediated its association with the total aggression score, anger score, hostility, and verbal and physical aggression. These findings imply that these specific personality traits (i.e., RS and self-compassion) may constitute specific aspects of the broader concept of borderline personality, which encompasses negative cognitions and emotions regarding self and others (American Psychiatric Association [APA], 2013). In other words, high RS and low self-compassion each translate into greater borderline personality features, which in turn relate to increased aggressive behavior. Indeed, studies have shown that individuals with borderline personality features are predominantly sensitive to interpersonal rejection and tend to be extremely reactive, displaying impulsive and aggressive behaviors in daily situations in which they feel excluded, abandoned, or rejected (Ayduk et al., 1999, 2008; Staebler et al., 2011). This supposition is strengthened by the differences that were found between the study groups: participants diagnosed with borderline personality disorder reported lower levels of self-compassion and higher levels of RS in comparison to their counterparts. Moreover, the fact that the mediation analyses were conducted while controlling for group (1 = borderline, 0 = control) serve as additional support to this assumption, since it reveals that even beyond the participants’ self-attribution to groups, borderline features mediate the relationships between self-compassion and RS, and aggressive behavior. Aside to the above, it should be noted that the competing model in which self-compassion and RS mediate the relationship between borderline features and aggression was not supported by our data.

These findings imply that self-compassion and RS explain variance in borderline personality features, and support a dimensional conceptualization of BPD. It may also support the notion of a developmental pathway (Bradley and Westen, 2005), by which RS and low self-compassion are personality tendencies formed within relationships with primary caregivers, that may then organize to varying degrees into more complex personality configurations, such as borderline personality disorder. However, this assumption needs further empirical validation.

According to our findings, self-compassion and RS are negatively associated. We found that the more individuals are kind to themselves, the less they are prone to anxiously expect, readily perceive, and intensely react to rejection. As this finding is correlative, it is impossible to determine its causal direction. However, there are findings indicating that self-compassion is associated with lower levels of negative emotions in distressful interpersonal situations (Leary et al., 2007). Specifically, the second component of self-compassion (i.e., common humanity) is considered to account for the ability of people to recognize negative experiences such as loss, rejection, or humiliation as part of common human experience, and so adaptively cope with them more effectively (Neff, 2003b; Allen and Leary, 2010). The relationship between the ability to be kind to oneself and the experience of self in the interpersonal context needs to be further studied.

The present study has several limitations. First, the sample size was limited. Therefore, the conclusions of the present study should be taken cautiously. Due to the small sample size, we conducted the mediation analysis on both groups pooled together. However, this might introduce deviations in the obtained results., Future studies should examine the replication of our findings in a larger sample. A larger sample will also allow to examine the role of gender as a moderating variable of the mediation. In addition, the borderline group was based on self-reported diagnosis, as other researchers have done in similar studies (e.g., Zvi and Elaad, 2018). Participants in this group were identified as members in a community supportive group of borderline personality patients and reported being diagnosed by a mental health professional. Future studies are needed to replicate these findings with a clinical sample, as well as with other than borderline personality configurations, in order to investigate the model’s specificity. Finally, this study is cross-sectional with all analyses based on data collected concomitantly, producing correlational findings. Thus, as noted, it is impossible to draw conclusions about causation.

In sum, the present study shows that RS and self-compassion are associated with borderline personality features, supporting the notion that borderline personality features are important in understanding the relationship between self-compassion and aggressive behavior, as well as between RS and aggressive behavior. The findings of the current study contribute to raising awareness of the relationships found in the study. They can help therapists find appropriate ways to deal with aggression, even with regard to personality traits, beyond observed behavior. Interventions with borderline patients should set as goals the reduction of RS and strengthening of self-compassion. These interventions may lessen borderline cognitions and emotions, and, therefore, decrease patient proneness to hostile and aggressive responses.

## Data Availability Statement

All datasets generated for this study are included in the article/supplementary material.

## Ethics Statement

This study was carried out in accordance with the recommendations of Ariel University Institutional Review Board (IRB). Written informed consent was obtained from all subjects. All subjects gave written informed consent in accordance with the Declaration of Helsinki. The protocol was approved by the Ariel University IRB.

## Author Contributions

Both authors were involved in planning, data analyses, and the preparation of the manuscript.

## Conflict of Interest

The authors declare that the research was conducted in the absence of any commercial or financial relationships that could be construed as a potential conflict of interest.

## References

[B1] Allen A. B. Leary M. R. (2010). Self-Compassion, stress, and coping. *Soc. Personal. Psychol. Compass* 4 107–118. 10.1111/j.1751-9004.2009.00246.x PMC2914331 20686629

[B2] Allen B. Cramer R. J. Harris P. B. Rufino K. A. (2013). Borderline personality symptomatology as a mediator of the link between child maltreatment and adult suicide potential. *Arch. Suicide Res.* 17 41–51. 10.1080/13811118.2013.748413 23387402

[B3] American Psychiatric Association [APA], (2013). *Diagnostic and Statistical Manual of Mental Disorders* (5th ed). Arlington, VA: American Psychiatric Publishing.

[B4] Ayduk O. Downey G. Testa A. Yen Y. Shoda Y. (1999). Does rejection elicit hostility in rejection sensitive women? *Soc. Cogn.* 17 245–271. 10.1521/soco.1999.17.2.245

[B5] Ayduk Ö. Gyurak A. Luerssen A. (2008). Individual differences in the rejection-aggression link in the hot sauce paradigm: the case of rejection sensitivity. *J. Exp. Soc. Psychol.* 44 775–778. 20228947 10.1016/j.jesp.2007.07.004 PMC2836513

[B6] Barnard L. K. Curry J. F. (2011). Self-compassion: conceptualizations, correlates, & interventions. *Rev. Gen. Psychol.* 15 289–303. 10.1037/a0025754

[B7] Baron R. M. Kenny D. A. (1986). The moderator-mediator variable distinction in social psychological research: conceptual, strategic, and statistical considerations. *J. Personal. Soc. Psychol.* 51 1173–1182. 10.1037/0022-3514.51.6.1173 3806354

[B8] Barry C. T. Loflin D. C. Doucette H. (2015). Adolescent self-compassion: associations with narcissism, self-esteem, aggression, and internalizing symptoms in at-risk males. *Personal. Individ. Differ.* 77 118–123. 10.1016/j.paid.2014.12.036

[B9] Berenson K. R. Gyurak A. Ayduk O. Downey G. Garner M. J. Mogg K. (2009). Rejection sensitivity and disruption of attention by social threat cues. *J. Res. Personal.* 43 1064–1072. 10.1016/j.jrp.2009.07.007 20160869 PMC2771869

[B10] Bowlby J. (1980). *Attachment and Loss: Vol. 3. Loss, Sadness, and Depression.* New York, NY: Basic.

[B11] Bradley R. Westen D. (2005). The psychodynamics of borderline personality disorder: a view from developmental psychopathology. *Dev. Psychopathol.* 17 927–957. 10.1017/s0954579405050443 16613425

[B12] Buss A. H. Durkee A. (1957). An inventory for assessing different kinds of hostility. *J. Consult. Psychol.* 21 343–349. 10.1037/h0046900 13463189

[B13] Buss A. H. Perry M. P. (1992). The aggression questionnaire. *J. Personal. Soc. Psychol.* 63 452–459. 10.1037//0022-3514.63.3.452 1403624

[B14] Carver C. S. (2014). Self-control and optimism are distinct and complementary strengths. *Personal. Individ. Differ.* 66 24–26. 10.1016/j.paid.2014.02.041

[B15] DeWall C. N. Twenge J. M. Gitter S. A. Baumeister R. F. (2009). It’s the thought that counts: the role of hostile cognition in shaping aggressive responses to social exclusion. *J. Personal. Soc. Psychol.* 96 45–59. 10.1037/a0013196 19210063 PMC2775524

[B16] Downey G. Feldman S. (1996). Implications of rejection sensitivity for intimate relationships. *J. Soc. Personal. Psychol.* 70 1327–1343. 10.1037/0022-3514.70.6.1327 8667172

[B17] Downey G. Feldman S. Ayduk O. (2000). Rejection sensitivity and male violence in romantic relationships. *Pers. Relatsh.* 7 45–61. 10.1177/0886260515609584 26467933 PMC6197802

[B18] Downey G. Lebolt A. Rincon C. Freitas A. (1998). Rejection sensitivity and children’s interpersonal difficulties. *Child Dev.* 69 1074–1091. 10.1111/j.1467-8624.1998.tb06161.x 9768487

[B19] Downey G. Mougios V. Ayduk O. London B. E. Shoda Y. (2004). Rejection sensitivity and the defensive motivational system: insights from the startle response to rejection cues. *Psychol. Sci.* 15 668–673. 10.1111/j.0956-7976.2004.00738.x 15447637

[B20] Feldman S. Downey G. (1994). Rejection sensitivity as a mediator of the impact of childhood exposure to family violence on adult attachment behavior. *Dev. Psychopathol.* 6 231–247. 10.1017/S0954579400005976 23438329

[B21] Hair F. H. Anderson R. E. Tatham R. L. Black W. C. (1995). *Multivariate Data Analysis.* Upper Saddle River: Prentice Hall.

[B22] IBM Corp, (2012). *IBM SPSS Statistics for Windows, Version 21.0*. Armonk, NY: IBM Corp.

[B23] In-Albon T. Suppiger A. Schlup B. Wendler S. Margraf J. Schneider S. (2008). Validität des Diagnostischen Interviews bei psychischen Störungen (DIPS für DSM-IV-TR) [Validity of the ‘Diagnostisches Interview bei psychischen Störungen (DIPS für DSM-IV-TR)’]. *Z. Klin. Psychologie* 37 33–42. 10.1026/1616-3443.37.1.33

[B24] Kohut H. (1977). *The Restoration of the Self.* New York, NY: International Universities Press.

[B25] Lawson D. M. Brossart D. F. (2013). Interpersonal problems and personality features as mediators between attachment and intimate partner violence. *Violence Vict.* 28 414–428. 10.1891/0886-6708.vv-d-12-00031 23862307

[B26] Leary M. R. Tate E. B. Adams C. E. Batts Allen A. Hancock J. (2007). Self-compassion and reactions to unpleasant self-relevant events: the implications of treating oneself kindly. *J. Personal. Soc. Psychol.* 92 887–904. 10.1037/0022-3514.92.5.887 17484611

[B27] Leary M. R. Twenge J. M. Quinlivan E. (2006). Interpersonal rejection as a determinant of anger and aggression. *Personal. Soc. Psychol. Rev.* 10 111–132. 10.1207/s15327957pspr1002_2 16768650

[B28] Mauricio A. M. Tein J. Y. Lopez F. G. (2007). Borderline and antisocial personality scores as mediators between attachment and intimate partner violence. *Violence Vict.* 22 139–157. 10.1891/088667007780477339 17479552

[B29] Morley R. H. (2015). Violent criminality and self-compassion. *Aggress. Violent Behav.* 24 226–240. 10.1016/j.avb.2015.05.017

[B30] Neff K. D. (2003a). Self-compassion: an alternative conceptualization of a healthy attitude toward oneself. *Self Identity* 2 85–102.

[B31] Neff K. D. (2003b). The development and validation of a scale to measure self-compassion. *Self Identity* 2 223–250. 10.1080/15298860309027 26979311

[B32] Neff K. D. (2016). The Self-Compassion Scale is a valid and theoretically coherent measure of self-compassion. *Mindfulness* 7 264–274. 10.1007/s12671-015-0479-3

[B33] Neff K. D. Beretvas S. N. (2013). The role of selfcompassion in romantic relationships. *Self Identity* 12 78–98. 10.1080/15298868.2011.639548

[B34] Neff K. D. Kirkpatrick K. L. Rude S. S. (2007). Self-compassion and adaptive psychological functioning. *J. Res. Personal.* 41 139–154. 10.1016/j.jrp.2006.03.004

[B35] Neff K. D. Pommier E. (2013). The relationship between self-compassion and other-focused concern among college undergraduates, community adults, and practicing meditators. *Self Identity* 12 160–176. 10.1080/15298868.2011.649546

[B36] Neff K. D. Vonk R. (2009). Self-compassion versus global self-esteem: two different ways of relating to oneself. *J. Pers.* 77 23–50. 10.1111/j.1467-6494.2008.00537.x 19076996

[B37] Preacher K. J. Hayes A. F. (2004). SPSS and SAS procedures for estimating indirect effects in simple mediation models. *Behav. Res. Methods Instrum. Comput.* 36 717–731. 10.3758/bf03206553 15641418

[B38] Reijntjes A. Thomaes S. Kamphuis J. H. Bushman B. J. De Castro B. O. Telch M. J. (2011). Explaining the paradoxical rejection-aggression link: the mediating effects of hostile intent attributions, anger, and decreases in state self-esteem on peer rejection-induced aggression in youth. *Personal. Soc. Psychol. Bull.* 37 955–963. 10.1177/0146167211410247 21632967

[B39] Renneberg B. Schmidt-Rathjens C. Hippin R. Backenstrass M. Fydrich T. (2005). Cognitive characteristics of patients with borderline personality disorder. *J. Behav. Ther. Exp. Psychiatry* 36 173–182. 15950176 10.1016/j.jbtep.2005.05.001

[B40] Rosenbach C. Renneberg B. (2014). Rejection sensitivity as a mediator of the relationship between experienced rejection and borderline characteristics. *Pers. Individ. Dif.* 69 176–181. 10.1016/j.paid.2014.05.032

[B41] Sansone R. A. Sansone L. A. (2012). Borderline personality and externalized aggression. *Innov. Clin. Neurosci.* 9 23–26. 22567607 PMC3342993

[B42] Shechory M. Weiss J. Weinstain R. (2013). Differentiating offenders by index offence and personality inventories: the characteristics of adult probationers in Israel. *Int. J. Offender Ther. Comp. Criminol.* 57 312–331. 10.1177/0306624x11428316 22116962

[B43] Staebler K. Helbing E. Rosenbach C. Renneberg B. (2011). Rejection sensitivity and borderline personality disorder. *Clin. Psychol. Psychother.* 18 275–283. 10.1002/cpp.705 21110407

[B44] Yarnell L. M. Neff K. D. (2013). Self-compassion, interpersonal conflict resolutions, and well-being. *Self Identity*, 12 146–159. 10.1080/15298868.2011.649545

[B45] Zessin U. Dickhäuser O. Garbade S. (2015). The relationship between self-compassion and well-being: a meta-analysis. *Appl. Psychol. Health Well-Being* 7 340–364. 10.1111/aphw.12051 26311196

[B46] Zvi L. Elaad E. (2018). Correlates of narcissism, self-reported lies, and self-assessed abilities to tell and detect lies, tell truths, and believe others. *J. Investig. Psychol. Offender Profiling* 15 271–286. 10.1002/jip.1511

